# Physiological consequences of prolonged static sedentary work among corporate desk employees: a systematic review with implications for the Indian occupational context

**DOI:** 10.3389/fpubh.2026.1859767

**Published:** 2026-07-29

**Authors:** Mohd Nauman Khan

**Affiliations:** Department of Design and Innovation, Faculty of Architecture and Ekistics, Jamia Millia Islamia, New Delhi, India

**Keywords:** Active Couch Potato effect, corporate desk workers, endothelial dysfunction, musculoskeletal disorders, occupational health, occupational sitting, sedentary behavior, systematic review

## Abstract

**Background:**

Corporate desk employees spend 6–10 h per working day in sustained static sitting, yet no systematic review has synthesized the multi-pathway physiological evidence for this population with reference to the Indian occupational context.

**Methods:**

Preferred Reporting Items for Systematic Reviews and Meta-Analyses (PRISMA) 2020-compliant systematic review. PubMed/MEDLINE, Scopus, Web of Science, and Cumulative Index to Nursing and Allied Health Literature (CINAHL) were searched (January 2012 to December 2025). Research question was framed using Population, Intervention (or Exposure), Comparator, Outcomes, and Study Design (PICOS). Eleven peer-reviewed sources were included and synthesized narratively by physiological pathway.

**Results:**

Included evidence reports WMSD prevalence up to 80.81% among office workers, with IL-6 proposed as a full mediator of the postural load to lower back disorder pathway (OR = 4.27; 95% CI: 1.50–12.10). Popliteal artery FMD declined from 7.1% to 2.8% after 3 h of uninterrupted sitting in a controlled laboratory study, through a shear stress mechanism. Sedentary time exceeding 10.6 daily hours is associated with 40–60% greater cardiovascular mortality risk, independent of physical activity compliance, consistent with the Active Couch Potato hypothesis.

**Conclusion:**

Prolonged static sitting is associated with harm across musculoskeletal, vascular, inflammatory, and cardiovascular pathways through mechanisms current ergonomic interventions do not fully address. Lower limb muscular inactivity appears to be the common upstream factor. Indian-specific occupational cohort studies are the primary evidence gap identified.

## 1 Introduction

India's corporate sector has moved a large working population into full-time desk-based employment. Information technology, financial services, banking, and consulting together employ millions of adults who sit for 6–10 h each working day. The physiological consequences of this occupational pattern are not well-integrated in the occupational health literature, particularly in the Indian context.

WHO defines sedentary behavior as waking activity at or below 1.5 metabolic equivalents in a sitting posture (1). The definition separates sedentary behavior from physical inactivity: a person can meet exercise guidelines and still accumulate substantial occupational sitting with independent physiological consequences. This separation matters clinically. Research examining the Active Couch Potato hypothesis—the proposition that sedentary time exerts health associations beyond what physical activity attenuates—suggests that exercise alone may not offset occupational sitting risk, though this remains under active investigation (2, 3).

India's cardiovascular disease burden adds contextual relevance. Government statistics report 32,457 cardiac fatalities in 2022, a 12% year-on-year rise, with a disproportionate share among younger working-age adults (4). These trends coincide with rapid growth in desk-based corporate employment and high reported occupational working hours (5). Direct causal linkages between desk work and these outcomes have not been established in Indian populations, but the structural features of Indian corporate employment—long hours, sedentary commuting, absent ergonomic regulation—make occupational sedentary exposure a relevant candidate exposure warranting attention.

Four physiological pathways have been independently characterized in the literature: musculoskeletal, vascular, inflammatory, and cardiovascular. No prior systematic review has integrated these pathways in a single synthesis examining their relevance to corporate desk workers and the Indian occupational context. The present review addresses this gap, drawing on peer-reviewed evidence, and discusses implications for occupational health policy and intervention design.

## 2 Methods

### 2.1 PICOS and study design

Systematic review conducted per PRISMA 2020 (6). Single-author design; absence of independent dual screening is a methodological limitation (see Section 4.1). Research question structured using PICOS: Population—adult corporate desk employees (≥18 years); Exposure—prolonged static occupational sitting (≥6 h per working day); Comparator—lower sedentary exposure or active conditions where reported; Outcomes—physiological consequences across four predefined domains (musculoskeletal, vascular, inflammatory, cardiovascular); Study designs—prospective cohorts, cross-sectional surveys, RCTs, laboratory experiments, nested case-controls, systematic reviews, and meta-analyses.

### 2.2 Eligibility

Included: peer-reviewed quantitative empirical studies or systematic reviews reporting physiological outcomes in adults in desk-based occupations; published in English, January 2012 to December 2025; extractable data for at least one of the four target pathways. Excluded: studies examining exclusively leisure-time sedentary behavior; animal studies, editorials, opinion pieces, and abstracts; studies treating sedentary behavior as a secondary covariate in clinical populations; insufficient methodological detail for quality appraisal. Indian national statistics and gray literature were used solely as contextual background; they were not treated as primary physiological evidence and were not subjected to study quality criteria.

### 2.3 Search and selection

Four databases were searched: PubMed/MEDLINE, Scopus, Web of Science, and CINAHL. Search string:

(“sedentary behaviour” OR “sedentary behavior” OR “prolonged sitting” OR “desk work” OR “office work”) AND (“musculoskeletal disorder” OR “endothelial dysfunction” OR “flow-mediated dilation” OR “inflammatory cytokine” OR “interleukin-6” OR “cardiovascular risk” OR “Active Couch Potato”) AND (“office worker” OR “desk worker” OR “corporate employee” OR “sedentary occupation”)

**Figure 1:**
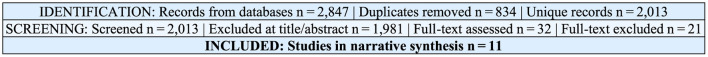
PRISMA 2020 flow diagram.

Filters: English language; human participants; January 2012 to December 2025. Search completed January 2026. Records deduplicated and screened against eligibility criteria using a structured checklist. Full texts retrieved for all records not clearly excluded. Exclusion reasons documented at full-text stage. Study selection is shown in Figure 1.

### 2.4 Data extraction and quality assessment

Data extracted by the author into a standardized form piloted on five studies: first author, year, country, design, sample size, exposure measure, outcomes, effect estimates, and quality rating. A cross-check pass was completed after initial extraction to reduce transcription error. Quality assessed using design-appropriate tools: NIH Quality Assessment Tool for observational studies; Cochrane RoB 2 for RCTs; AMSTAR-2 for included systematic reviews. Single-reviewer assessment is acknowledged as a limitation.

## 3 Results

### 3.1 Study selection

Two thousand eight hundred forty-seven records identified (PubMed: 1,124; Scopus: 847; Web of Science: 601; CINAHL: 275). After deduplication: 2,013 screened; 1,981 excluded at title/abstract stage (leisure-time only: 743; clinical populations: 512; animal studies: 219; insufficient physiological data: 507). Thirty-two full-text records assessed; 21 excluded (PA interventions without sedentary-specific outcomes: 5; insufficient methodology: 3; non-occupational populations: 2; data not disaggregated by exposure: 2; sources serving as background or contextual references rather than contributing extractable physiological evidence: 9). Eleven peer-reviewed sources met all eligibility criteria and were included in the final synthesis.

### 3.2 Characteristics of included studies

Eleven peer-reviewed sources were included: two nested case-control studies, one prospective cohort, one cross-sectional study, one laboratory RCT, one biomechanical study, four systematic reviews or meta-analyses, and one conceptual review. The conceptual review (2) was included because it provides the primary theoretical and mechanistic framework for the Active Couch Potato hypothesis central to the cardiovascular pathway synthesis; while not an empirical study, it constitutes a peer-reviewed scientific contribution directly relevant to the outcomes of interest. Countries represented: USA (*n* = 3), UK (*n* = 1), Canada (*n* = 1), China (*n* = 1), Australia (*n* = 1), Ireland (*n* = 1), multi-country (*n* = 3). Sample sizes ranged from 14 participants (laboratory RCT, Thosar et al.) to 89,530 participants (prospective cohort, Ajufo et al.). None of the included studies were conducted in India; all physiological evidence originates from non-Indian populations, substantially limiting the strength of India-specific conclusions drawn in this review. Study quality was predominantly moderate; two studies were rated at high risk of bias due to self-reported exposure and potential confounding by leisure-time physical activity. Table 1 summarizes all included sources.

**Table 1:** Key included studies.

References	Country	Design	*N*	Exposure	Pathway	Key finding
Elsawy et al. (7)	Multi	SR/MA	Multiple	NMQ/self-report	Musculoskeletal	WMSD prevalence 80.81%; neck 58.6%; LBP 52.5%; shoulder 37.4%
Dong et al. (8)	China	Nested case-control	NR (university employees)	REBA/OWAS ergonomic assessment	Musculoskeletal + Inflammatory	OR 4.27 (95% CI 1.50–12.10) for LBP; IL-6 full mediator of pathway
Thosar et al. (9)	USA	Laboratory RCT	14	Controlled 3h sitting protocol	Vascular	FMD: 7.1% to 2.8% after 3 h; shear stress mechanism confirmed by heating control
Ajufo et al. (13)	UK	Prospective cohort	89,530	Wrist accelerometry	Cardiovascular	>10.6 h/day: 40–60% greater CVD death risk; persists with PA compliance
Hamilton et al. (2)	USA	Conceptual review	N/A	N/A	Cardiovascular	Active Couch Potato hypothesis proposed; LPL mechanism characterized
Karuc et al. (10)	Multi	SR/MA of RCTs	Multiple	Controlled interruption protocols	Vascular	Sitting interruptions: FMD MD +1.74%; shear rate MD +7.58 s?^1^; blood flow MD +12.08 ml/min
Mac Eochagain et al. (11)	Ireland	Systematic review	Multiple	Self-report/observation	Intervention	Standing desk sustained use < 30% in most cohorts; rapid reversion to sitting
Freene et al. (3)	Australia	Cross-sectional	261	Self-report	Cardiovascular	Sedentary time independent of PA; consistent with Active Couch Potato hypothesis
Ridker et al. (12)	USA	Nested case-control	618	Serum IL-6 bioassay (Physicians' Health Study)	Inflammatory	IL-6 associated with elevated MI risk independent of conventional cardiovascular risk factors
Callaghan and McGill (14)	Canada	Biomechanical study	Laboratory	Controlled standing posture	Intervention comparison	Prolonged standing associated with elevated lumbar paraspinal load; comparable burden to prolonged sitting
Shrestha et al. (15)	Multi	Cochrane SR	Multiple RCTs	Various workplace interventions	Intervention	Workplace sitting interventions show limited sustained behavioral compliance; adherence declines substantially within weeks

SR/MA, systematic review/meta-analysis; NMQ, nordic musculoskeletal questionnaire; FMD, flow-mediated dilation; LBP, lower back pain; PA, physical activity; LPL, lipoprotein lipase; CVD, cardiovascular disease; MD, mean difference.

### 3.3 Musculoskeletal pathway

Elsawy et al. reported WMSD prevalence of 80.81% across office worker cohorts, with site-specific rates of 58.6% (neck), 52.5% (lower back), and 37.4% (shoulders) (7). These figures derive from predominantly cross-sectional studies using the Nordic Musculoskeletal Questionnaire; recall bias and sampling variation likely affect absolute values. Mechanically, sustained sitting produces lumbar flexion that elevates intervertebral disc pressure and impedes oxygen delivery to avascular spinal tissue. Neck pain arises through sustained extensor muscle activation during screen use.

Dong et al. added an inflammatory dimension to the musculoskeletal picture (8). In a nested case-control study of university employees, high static postural load (≥70% of working time in constrained postures by validated ergonomic assessment) was associated with significantly elevated serum IL-6, TNF-α, and IL-1β. Mediation analysis found that IL-6 accounted for the full indirect pathway from postural load to lower back disorder incidence (adjusted OR = 4.27; 95% CI: 1.50–12.10; *p* = 0.007). The observational design of this and related studies limits causal inference; reverse causality cannot be excluded.

### 3.4 Vascular pathway

Thosar et al. showed that 3 h of uninterrupted sitting reduced popliteal artery FMD from 7.1% to 2.8% in a controlled laboratory protocol (9). When lower limb shear stress was maintained experimentally through local heating, FMD decline was attenuated, confirming shear stress elimination as the mechanism rather than a correlate. FMD is an accepted surrogate marker of endothelial function with associations with future cardiovascular events, though extrapolation from acute laboratory conditions to chronic workplace exposure is a recognized limitation.

A meta-analysis of RCTs (10) found that sitting interruptions significantly improved shear rate (MD: +7.58 s^−1^), FMD (MD: +1.74%), and peripheral blood flow (MD: +12.08 ml/min) relative to uninterrupted sitting. Vascular impairment appears reversible with frequent interruption; however, real-world adherence to voluntary interruption protocols is consistently poor (11).

### 3.5 Inflammatory pathway

Beyond the musculoskeletal mediation finding, Dong et al. documented that high static postural load elevated TNF-α and IL-1β in workers without pre-existing inflammatory conditions, suggesting that occupational postural load may be a stimulus for systemic cytokine elevation (8). Theoretical models propose that prolonged muscular inactivity reduces secretion of anti-inflammatory myokines (IL-10, IL-15, adiponectin), shifting systemic cytokine balance toward pro-inflammatory states independently of local tissue stress. Direct empirical confirmation of this myokine mechanism in occupational populations is limited. Prospective cohort studies report independent associations between elevated IL-6 and long-term cardiovascular event risk (12), providing a plausible link between occupational inflammatory exposure and cardiovascular outcomes.

### 3.6 Cardiovascular pathway

Ajufo et al. analyzed accelerometry data from 89,530 UK Biobank participants over 8 years (13). Sedentary time exceeding 10.6 h per day was associated with 40–60% greater risk of heart failure and cardiovascular death. This association persisted in participants meeting WHO physical activity recommendations, which is consistent with the Active Couch Potato hypothesis (2, 3). This hypothesis proposes that LPL activity in skeletal muscle—governing plasma triglyceride clearance—is regulated by local muscular contraction during the day, not restored by post-work exercise alone, and that GLUT4 expression follows a similar local-activity-dependent pattern. These mechanisms, if confirmed in occupational cohorts, would explain why exercise may not fully offset sedentary sitting risk. The dose-response threshold of 10.6 h is not definitive; the relationship remains dose-dependent with ongoing investigation into precise thresholds. The Active Couch Potato concept is an evidence-supported hypothesis, not a settled scientific conclusion.

## 4 Discussion

Four physiological pathways converge on a common upstream factor: the absence of lower limb muscular activity during sustained sitting. This factor eliminates venous pump function, reduces arterial shear stress, suppresses anti-inflammatory myokines, and removes the contractile stimulus for LPL and GLUT4 activity. The convergence matters because it means that interventions targeting spinal posture alone—however well-designed—do not address the circulatory and metabolic mechanisms through which the most serious long-term consequences appear to operate.

Current ergonomic responses are designed around postural correction. Ergonomic chairs reduce disc pressure and muscular load but do not restore lower limb circulatory activity. Standing desks substitute one static posture for another—prolonged standing carries its own biomechanical costs including elevated lumbar paraspinal load and lower limb venous pooling (14)—and are associated with sustained use rates below 30% in real workplace settings within weeks of deployment (11). Wearable reminders and digital interruption tools theoretically align with the shear stress and LPL evidence but depend on behavioral compliance that available adherence data do not support at scale (15). These limitations do not mean ergonomic intervention is without value—postural optimization does reduce mechanical musculoskeletal load—but they suggest that postural intervention alone is insufficient against the multi-pathway evidence base. Future design research might usefully explore passive, built-in approaches to generating lower limb muscular activation during sitting without requiring behavioral effort. This is a hypothesis for future investigation rather than a validated approach; no intervention trials demonstrating the superiority of such designs over conventional ergonomic seating have been published to date.

The Indian context adds structural reasons to treat this problem as a priority. High reported working hours, sedentary urban commuting, and absent ergonomic standards under the Occupational Safety, Health and Working Conditions Code 2020 mean that total daily sedentary exposure for many Indian corporate workers likely exceeds the 10.6-h threshold associated with elevated cardiovascular mortality in the Ajufo et al. data. India's cardiovascular disease burden, including a disproportionate share of events in young working-age adults, is contextually consistent with these associations, though direct Indian occupational cohort data establishing these linkages do not exist. The Indian contextualization in this review is inferential, drawing on structural and epidemiological parallels rather than direct evidence—a limitation the title revision reflects.

The Active Couch Potato hypothesis has important implications for public health messaging if confirmed. Recommendations centered on post-work exercise may be insufficient if sedentary behavior and physical inactivity operate through partially distinct molecular pathways. This would mean the workstation, not the gym, is the necessary site of intervention. However, this conclusion rests on population-level observational data with residual confounding risk and proposed mechanisms not yet directly confirmed in occupational populations. Further RCT and cohort evidence is needed before translating the hypothesis into firm policy.

### 4.1 Limitations

Single-author design is the principal methodological limitation. All screening, extraction, and quality assessment were conducted by one reviewer without independent verification; inter-rater reliability metrics cannot be reported. None of the 11 included sources were conducted in India; all physiological evidence originates from non-Indian populations, making India-specific conclusions inferential rather than evidential. Cross-sectional designs in the musculoskeletal evidence preclude causal inference. Self-reported sedentary exposure introduces recall bias. The Active Couch Potato hypothesis has not been directly tested in occupational desk worker cohorts. Eleven included sources across four pathways is a limited evidence base; the synthesis should be interpreted as exploratory and directional rather than definitive.

## 5 Conclusion

Prolonged occupational sitting is associated with harm across musculoskeletal, vascular, inflammatory, and cardiovascular pathways in the published evidence. Lower limb muscular inactivity is the proposed common proximate mechanism, and existing ergonomic interventions do not adequately address it. The Active Couch Potato hypothesis suggests that physical activity outside work may not fully offset these risks, though the evidence base remains observational. The Indian corporate context, characterized by high working hours and absent ergonomic regulation, creates conditions in which these associations are structurally relevant; confirming them requires Indian-specific occupational cohort data, which represents the primary evidence gap this review identifies. Future research priorities include prospective Indian occupational cohort studies using objective exposure measurement, RCTs of passive design interventions targeting lower limb circulatory activation, and regulatory modeling for occupational sedentary exposure thresholds.

## Data Availability

The original contributions presented in the study are included in the article/supplementary material, further inquiries can be directed to the corresponding author.
